# Reviews and Notices

**Published:** 1886-10-30

**Authors:** 


### Reviews and Notices.

### CONVALESCENT HOMES FOR ASYLUM PATIENTS.

There is no department of medicine in which more progress has been made than in the care and cure of the insane. Lunatic asylums, at no very remote period, were abodes of frightful misery and horror, such as we cannot now picture to the imagination. Insanity was not understood by medical men, much less by the non-professional attendant; and where ignorance and fear were the guiding spirits, it was no wonder that cruelty and despair often followed in their train. Mental disorder, in all its forms and varieties, is now an understood and recognised disease. The object of treatment is not repression, but cure, and the results so frequently obtained completely justify the methods of modern times. It is generally believed that there is a kind of ideal completeness about the means and methods available for the treatment of mental disorders. That is no doubt correct so far as the well-to-do classes are concerned, but by no means so in the case of the poor. In regard to the treatment of the actually insane, the county asylums are in the very forefront of the onward march; but when treatment has had its legitimate result in the restoration of the patient to sanity, these asylums, through no fault of their own, are not able to add the touch of completeness to their beneficent work.

In an exceedingly practical and judicious pamphlet,* the Rev. H. Hawkins, Chaplain to the Colney Hatch Asylum, calls attention to a much-needed aid towards the complete cure of those who have for a time been mentally disabled. Mr. Hawkins's experience goes to show that patients who have recovered under the care and control of the asylum system, are often seriously checked in their onward progress by being too suddenly thrown into the midst of the harassing struggle for existence which is the daily lot of the poor. The mind which has but recently recovered its balance, is in no condition to sustain the various anxieties and worries of a poor man's home. Hence arise long periods of extreme unhappiness and depression, with frequent relapses into mental aberration, and an early return to the asylum. Very much of this grievous trouble might be prevented, Mr. Hawkins thinks, by the establishment of convalescent homes for those who are so far recovered as no longer to require the restraints of a regular asylum.

The idea is probably not new, but of its great and urgent importance there can be no doubt. The convalescent home should be in no sense an asylum, but a place absolutely free from restraint, where the patient might take up again *gradually* those duties and responsibilities which, if *suddenly* resumed, might prove a burden too heavy to be borne. No one familiar with the difficulties of maintaining existing charitable institutions, would lightly

* Reprinted from *Journal of Mental Science.*

encourage the founding of new ones; but in the progress of benevolence there can be no pause, and as new needs arise new impulses and new efforts must be developed to meet them. Mr. Hawkins's arguments are based on the sure ground of real and serious need, and in this country at any rate, it has never been known that the really distressed have been long left without succour. The scheme is eminently worthy of the thoughtful considera-tion of sober and duty-loving philanthropists.

---

*A Handbook on Nursing.* By CATHERINE JANE WOOD. Cassell & Co., Limited, London, Paris, New York, and Melbourne. Price 1s. 6d., or 2s. cloth.

THERE is no lack of books on nursing, though few of them reach any high standard of merit. Mrs. Catherine Jane Wood's manual can hardly be placed among the more meritorious few. Mrs. Wood writes with authority and definiteness. She shows unmistakably that she has a practical acquaintance with her subject, and that, of course, is one of the primary conditions of success in a teacher. But mere knowledge of a subject never made an author and never will. There is in this handbook a great deal too much of everything: too much philoso-phising, too much preaching, too much repetition, and too much letter-press. Nursing is a simple subject, and should be taught in a simple, natural way. The teacher or writer, for the most part, addresses simple people, and should never forget the fact. Mrs. Wood occupies as much space in telling us how to nurse as Whateley gives to the whole subject of Political Economy. If every topic on which man desires information were dealt with at this disproportionate length, a thousand life-times would not suffice to make him moderately acquainted with the useful literature of the world. Mrs. Wood would be well-advised to cut down the volume to a quarter of its size, to have it carefully read, with the view to its syntax, by a literary expert, and to make it simple and kindly, rather than dogmatic, in style. The publishers have done their best to make the book acceptable, and both the paper and type are excellent.

---

*A Manual of Urine Testing.* By K. M. HEANLEY. London: H. K. Lewis.

THIS little book is intended as a guide for matrons, nurses, and probationers. As such it will doubt-less be found useful. The tests and the proper method of carrying them out are clearly given, and may be easily understood by a novice. We think the nurses might have been spared the re-marks upon the manner of the formation of the constituents for which they are testing. This is beyond the scope of such a book, and the few and somewhat crude remarks made will rather tend to confuse than enlighten the readers. It is, however, an honest little book, by a practical nurse who knows her business, and who desires that others should know it too. We welcome the volume also because it is, so far as we are aware, the first work ever written by a Cottage Hospital Matron.

